# Computational Biology in the Discovery of Biomarkers in the Diagnosis, Treatment and Management of Cardiovascular Diseases

**Published:** 2024-09-05

**Authors:** Irene Batta, Ritika Patial, Ranbir C. Sobti, Devendra K. Agrawal

**Affiliations:** 1 Bothell High School, Bothell, Washington, USA; 2 Centre for Systems Biology and Bioinformatics, Panjab University, Chandigarh, India; 3 Department of Biotechnology, Panjab University, Chandigarh, India; 4 Department of Translational Research, Western University of Health Sciences, Pomona, California, USA

**Keywords:** Biomarkers, Cardiovascular diseases, Computational biology, Epigenetic biomarkers, Metabolic biomarkers, Natriuretic peptide, Troponin

## Abstract

Cardiovascular diseases are the leading cause of mortality worldwide, with a disproportionately high burden in low- and middle-income countries. Biomarkers play a crucial role in the early detection, diagnosis, and treatment of cardiovascular diseases by providing valuable insights into the normal and abnormal conditions of the heart and vascular system. The biomarkers derived from the cells and tissues can be identified and quantified in the blood and other body fluids and in tissues. Changes in their expression level under a pathological condition provide clinical information on the underlying pathophysiology that could have predictive, diagnostic, and prognostic value in the treatment of a disease process, and therefore incorporated in clinical guidelines. This enhances the effectiveness of biomarkers in risk stratification and therapeutic decisions in personalized medicine and improvement in patient outcomes. Biomarkers could be protein, carbohydrate, or genome-based and may also be derived from lipids and nucleic acids. Computational biology has emerged as a powerful discipline in biomarker discovery, leveraging computational techniques to identify and validate biological markers for disease diagnosis, prognosis, and drug response prediction. The convergence of advanced technologies, such as artificial intelligence, multi-omics profiling, liquid biopsies, and imaging, has led to a significant shift in the discovery and development of biomarkers, enabling the integration of data from multiple biological scales and providing a more comprehensive understanding of the complex signaling and transcriptional networks underlying disease pathogenesis. In this article, we reviewed the role of computational biology integrated with genomics, proteomics, and metabolomics, together with machine learning techniques and predictive modeling and data integration in the discovery of biomarkers in cardiovascular diseases. We discussed specific biomarkers, including epigenetic, metabolic, and emerging biomarkers, such as extracellular vesicles, miRNAs, and circular RNAs, and their role in the pathophysiology of the heart and vascular diseases

## 1. Introduction

Cardiovascular diseases are the leading cause of mortality worldwide, with an estimated 17.3 million deaths annually, a figure projected to rise to 23.6 million by 2030 [1,2]. The burden of these diseases is disproportionately high in low- and middle-income countries, accounting for approximately 80% of the 20.5 million CVD-related deaths in 2021 [3]. The pathophysiology of cardiovascular diseases is complex, often involving a combination of genetic, epigenetic, and lifestyle factors [4-8]. These disorders, which affect the heart and blood vessels, constitute a global public health concern. The range of cardiovascular diseases includes coronary heart disease, cerebrovascular disease, peripheral arterial disease, rheumatic heart disease, congenital heart disease, and deep vein thrombosis with pulmonary embolism. Many mediators are involved at various stages of the underlying pathophysiology in these disorders [9-15].

For the early detection, diagnosis, and treatment of cardiovascular diseases (CVDs), biomarkers play an important part. Biomarkers, such as proteins, carbohydrate, peptides, and microRNAs, offer valuable insights into the normal and abnormal conditions of the heart and vascular system, allowing for timely and precise medical interventions [16-20]. For example, the use of high-sensitivity troponin (hs-cTn) has transformed the diagnosis of acute coronary syndrome (ACS) by providing superior sensitivity and negative predictive value despite ongoing challenges related to specificity and standardization [16]. Moreover, biomarkers like C-reactive protein (CRP), troponin I or T, creatine kinase (CK-MB), B-type natriuretic peptide (BNP), and myoglobin (Mb) are crucial for monitoring and treating CVDs, offering quick detection, affordability, sensitivity, and selectivity through advanced biosensing methods [17-20]. New biomarkers such as triggering receptor expressed on myeloid cells (TREM-1), sirtuins, oncostatin M receptor, danger associated molecular patterns (DAMPs), extracellular vesicles and circular RNAs (circRNAs) are also gaining recognition for their potential in early diagnosis and targeting for treatment, reflecting dynamic changes in the cardiovascular system under various pathological conditions [21-27].

**Figure 1:**
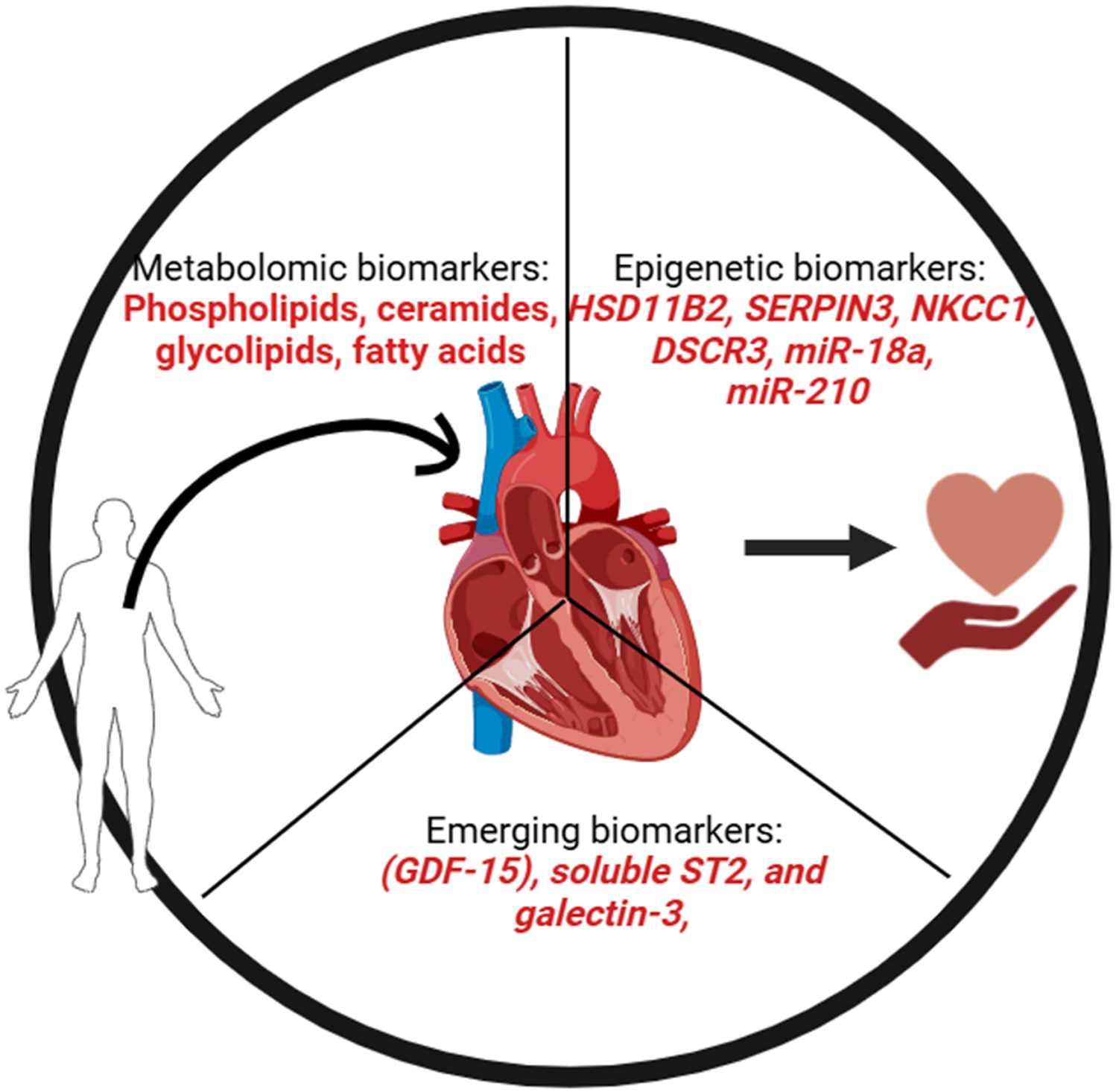
The schematic diagram represents known biomarkers in the pathophysiology of cardiovascular diseases.

Integrating genomics, proteomics, and molecular pathology has broadened the range of biomarkers, enhancing their effectiveness in personalized medicine and improving patient outcomes by customizing treatments to individual requirements [28-31] (Figure 1). Furthermore, the advancement of implantable microelectrodes using 3D printing techniques presents a promising tool for electrochemically detecting biomarkers, aiding in early diagnosis and personalized treatment approaches for CVDs and neurodegenerative diseases [32]. The utility of biomarkers extends beyond diagnosis to encompass risk assessment, therapy selection, disease progression, and recurrence monitoring, thus playing a vital role in the comprehensive management of CVDs [33,34]. For example, biomarkers such as suppression of tumorigenicity 2 protein (ST2), growth differentiation factor 15 (GDF-15), and galectin-3 (Gal3) have been investigated for their potential to enhance ACS management by reflecting myocardial injury, neurohormonal activation, inflammation, and thrombosis [18,19].

## 2. Biomarkers Provide Insights into Disease Pathophysiology

Biomarkers play a crucial role in understanding the pathophysiology of cardiovascular diseases (CVDs) and greatly assist in risk stratification and therapeutic decisions [2-4]. These biological indicators, including enzymes, proteins, peptides, and non-coding RNAs, offer additional prognostic details with significant clinical results and can be incorporated into tools supporting decisions for patients with acute and chronic cardiovascular conditions [9-17]. For example, cardiac biomarkers such as troponins and natriuretic peptides are vital for promptly diagnosing and managing acute coronary syndromes (ACS) and heart failure, respectively, enabling timely interventions during the critical period [16-19]. The utilization of biomarkers goes beyond diagnosis; they also play a key role in monitoring the response to therapy, evaluating prognosis, and stratifying risk, all of which are crucial for personalized or precise cardiology [16-19].

Emerging biomarkers, such as microRNAs and other genetic and metabolic indicators, have been discovered, providing deeper insights into the pathophysiological processes of CVDs and enabling more precise risk stratification and prediction of cardiovascular events [8,13,22]. The inclusion of biomarkers in risk prediction models, such as the ABC (Age, Biomarkers, Clinical history) risk score, has demonstrated superior performance compared with traditional risk scores, enhancing the accuracy of risk assessment and guiding the appropriate intensity of medical treatment to decrease future CVD risk. Moreover, biomarkers such as B-type natriuretic peptide and high-sensitivity cardiac troponin I show potential for enhancing risk prediction and secondary prevention of CVDs [18,19].

The clinical usefulness of biomarkers is also evident in their incorporation into clinical guidelines, which advocates for their use in diagnostic, prognostic, and therapeutic contexts, although factors such as cost, and assay variability need to be considered. Biomarkers are crucial for tracking disease advancement and evaluating therapeutic approaches in coronary artery disease, thus refining treatment strategies and lowering healthcare expenses. The identification of newer biomarkers, such as soluble sources of tumorigenicity 2 (sST2) and galectin-3, further boosts the ability to forecast, diagnose, and manage various cardiovascular conditions, showing the intricate interaction of inflammatory, genetic, neurohormonal, and biochemical elements in cardiac pathophysiology. Additionally, lipid biomarkers and inflammation markers are emphasized in the guidelines for the primary prevention of atherosclerotic cardiovascular disease (ASCVD), aiding in shared decision-making and potentially enhancing clinical outcomes.

## 3. Computational Biology in Biomarker Discovery

Computational biology has emerged as a powerful discipline that integrates various scientific fields, including biology, computer science, and mathematics, to tackle complex problems in life sciences. One such application of computational biology is in the field of biomarker discovery, in which researchers leverage computational techniques to identify and validate biological markers that can be used for disease diagnosis, prognosis, and drug response prediction [35-38]. The convergence of advanced technologies such as artificial intelligence, multi-omics profiling, liquid biopsies, and imaging has led to a significant shift in the discovery and development of biomarkers [35-38]. These technological advancements have enabled the integration of data from multiple biological scales, providing a more comprehensive understanding of the complex signaling and transcriptional networks that underlie disease pathogenesis [35].

## 4. Computational Biology Integrates Various 'Omic' Data (Genomics, Proteomics, Metabolomics) to Identify Potential Biomarkers

**Figure 2:**
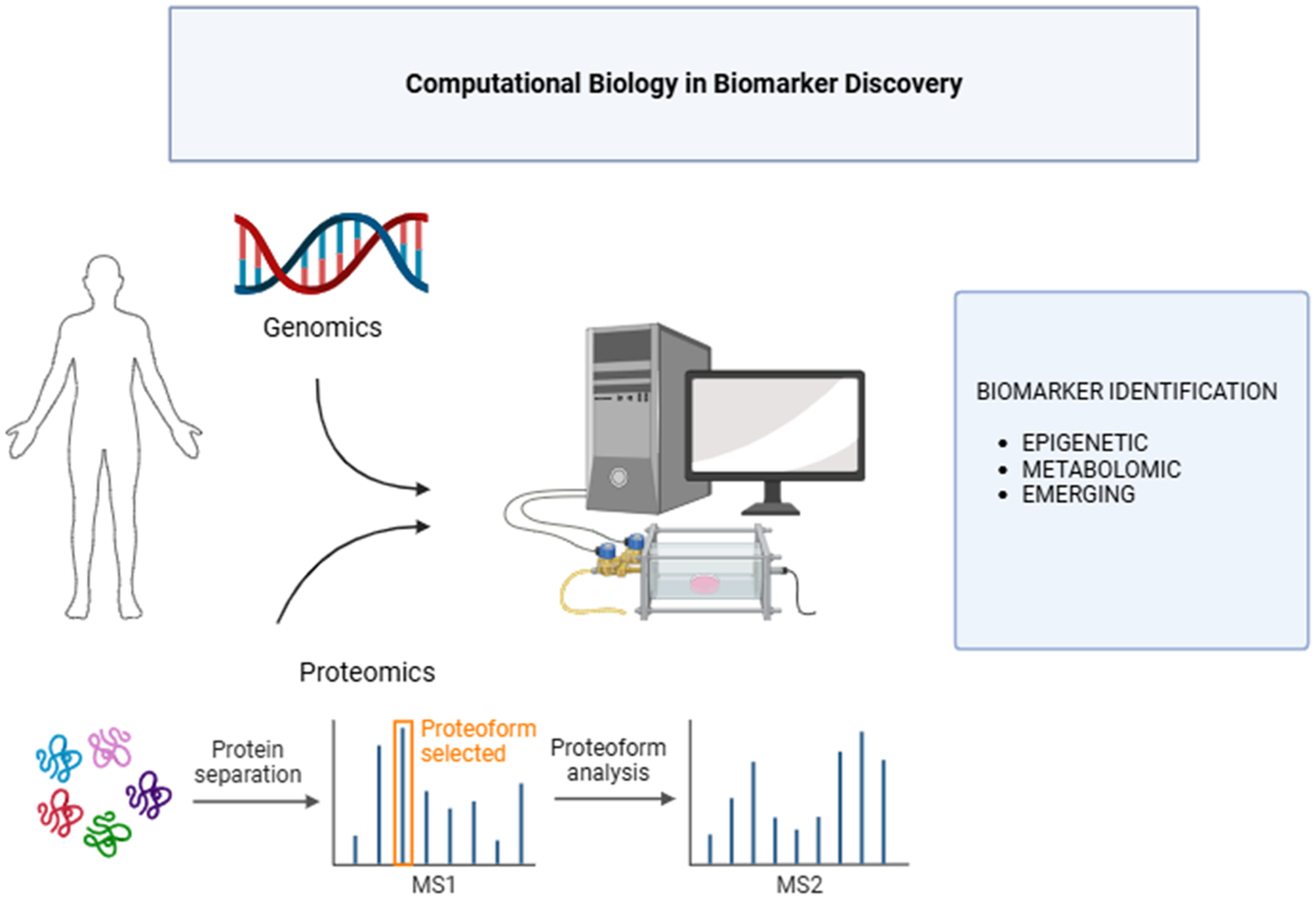
Computational biology integrates genomics and proteomics data to find significant biomarkers associated with diseases.

Computational biology has significantly progressed the discovery of potential biomarkers for cardiovascular diseases (CVDs) by integrating various 'omic' data, such as genomics, proteomics, and metabolomics as shown in Figure 2. This integrated approach enables a comprehensive understanding of the molecular mechanisms underlying CVDs. For example, metabolomics has been utilized to analyze serum metabolic profiles, pinpointing specific metabolites such as arginine, hypoxanthine, and acetylcarnitine, which can differentiate between coronary heart disease (CHD) and myocardial infarction (MI) with precision [39-44].

Proteomics has demonstrated substantial clinical value in CVD diagnosis by identifying protein biomarkers through systematic reviews and integrated analyses, thereby boosting the sensitivity and specificity of diagnostic models [34, 45]. Systems biology strategies further enhance this field by charting intricate networks of molecular functions throughout the genome, transcriptome, proteome, and metabolome, thus advancing our knowledge of heart diseases and facilitating the discovery of new biomarkers and treatments [28-31]. Furthermore, the merging of artificial intelligence and machine learning techniques with conventional statistical methods has facilitated the recognition of significant transcriptomic biomarkers, achieving an accuracy of up to 96% in predicting CVDs. This method enhances early detection and supports tailored treatment plans [41]. Moreover, merged mass spectrometry-based workflows combining metabolomics and proteomics have been employed to identify and quantify circulating small molecules and proteins and identify potential biomarkers associated with CVDs in patients with end-stage kidney disease (ESKD) [43,45,46]. These unified 'omic' methodologies, supported by computational biology, establish a sturdy framework for identifying and validating biomarkers, ultimately boosting cardiovascular disease diagnosis, prognosis, and treatment.

## 5. Machine Learning Techniques Such as Clustering and Support Vector Machines (SVMs) are Commonly Used for Biomarker Discovery

Machine learning techniques, such as clustering and support vector machines, play a crucial role in identifying biomarkers for cardiovascular diseases (CVDs) owing to their ability to manage high-dimensional data and reveal intricate patterns. Clustering approaches, such as hierarchical and k-means clustering, are utilized to cluster similar data points, aiding the identification of distinct patient subgroups based on their biomarker profiles [42-44]. This process helps discover new biomarkers that reflect specific CVD phenotypes. For example, clustering can analyze extensive datasets from real-world studies, facilitating the identification of patient subgroups with unique risk profiles, and assisting in personalized treatment strategies [41]. On the other hand, support vector machines are robust supervised learning algorithms that can categorize data by determining the optimal hyperplane that separates various classes. In the realm of CVD biomarker discovery, support vector machines have been employed to rank transcriptomic features and identify significant biomarkers by analyzing gene expression data [41,47]. For example, a study that assessed the top ten percent of important biomarkers using support vector machines in combination with other machine learning classifiers showed good accuracy in differentiating between patients with CVD and healthy persons [41]. Moreover, support vector machines have been used alongside surface-enhanced Raman spectroscopy (SERS) to classify intricate biofluid samples, showing high accuracy in distinguishing between physiological and pathological states based on the presence of key CVD biomarkers [47].

## 6. Predictive Modeling and Data Integration

Predictive modeling involves the integration of different types of data to screen, diagnose, or prognosticate CVDs. Predictive modelling of cardiovascular diseases (CVDs) requires incorporating various data types, such as demographic, clinical, biometric, and genetic information, to improve the accuracy of screening, diagnosis, and prognosis. Different machine learning and deep learning methods are used to handle these complex data, ensuring strong and dependable predictions. For example, Miao employed K-means++ clustering, Principal Component Analysis (PCA), and Logistic Regression to preprocess and decrease data dimensionality, thereby enhancing the accuracy and reliability of CVD predictions [48]. Similarly, Babalola et al. [49] investigated the use of support vector machines, ensemble learning, and artificial neural networks (NNs) to capture the intricate relationships between clinical and biometric data, achieving high accuracy and area under the curve values for predicting CVD events [49]. Ejiyi and team stress the significance of deep learning models combined with advanced data mining techniques, achieving high accuracies using classifiers like Extra Trees, Random Forest, AdaBoost, and XG-Boost [50]. Petreska's research showcases the superior performance of machine learning models compared to traditional statistical methods in early CVD detection, utilizing extensive datasets encompassing demographic, lifestyle, and health status parameters [51]. Shanthi et al. [52] demonstrated the effectiveness of Logistic Regression, K-Nearest Neighbor (KNN) Classifier, and Gaussian Naive Bayes (Gaussian NB) for predicting heart disease, with Gaussian NB consistently performing well across various datasets [52]. Several investigators further endorsed the use of multiple machine learning algorithms, including logistic regression, support vector machine, naive Bayes, random forest, and KNN, to effectively classify and predict CVDs [39-44]. Usova and colleagues discuss integrating "omics" data like genomics with artificial intelligence/machine learning strategies to enhance the assessment of atherosclerosis-related CVDs, using methods like Integrative Network Fusion for validation [54]. Ogunpola and team tackle the challenge of imbalanced datasets in CVD prediction, optimizing models like XGBoost to achieve remarkable accuracy and precision [55]. Exploring the use of electrocardiography (ECG) data in predictive models, advanced deep learning techniques, such as the multi-label semi-supervised model (ECGMatch), have been developed to identify multiple CVDs simultaneously, even with limited supervision. Finally, Haider and colleagues stressed the importance of automated systems in medical diagnosis, particularly in resource-limited settings, employing various machine learning algorithms with hyperparameter tuning to effectively predict CVDs [56].

Together, these studies highlight the critical role of integrating diverse data types and utilizing advanced machine learning and deep learning techniques to enhance the predictive modelling of CVDs, ultimately aiding in early intervention, personalized treatment strategies, and improved patient outcomes.

## 7. Specific Biomarkers and their Roles

Biomarkers are measurable indicators of some biological state or condition and can provide valuable insights into the underlying pathophysiology of cardiovascular diseases. In the context of cardiovascular diseases, biomarkers can be used to assess the risk of developing the disease, detect the presence of the disease, monitor the progression of the disease, and guide treatment decisions. Table 1 is compiled from many published reports and provides a list of the biomarkers based on different pathophysiological processes of heart diseases.

### 7.1 Cardiac Troponins and Natriuretic Peptides

Cardiac troponins (cTnI and cTnT) and natriuretic peptides (NPs) are widely recognized biomarkers of myocardial injury and dilation, respectively, because of their specific functions and diagnostic capabilities in cardiovascular diseases (CVD). When myocardial cells are damaged, cardiac troponins are released into the bloodstream, making them highly specific indicators of myocardial injury, such as acute myocardial infarction [57]. High-sensitivity tests for troponins can even identify minor myocardial injuries, which are essential for early detection and risk assessment in various clinical scenarios, including COVID-19, where elevated troponin levels have been associated with higher mortality rates and the need for mechanical ventilation [58-60]. Troponins also play a significant role in evaluating myocardial injury in hospitalized COVID-19 patients, with their increase indicating underlying pathological processes that can be further explored through cardiovascular magnetic resonance imaging [61]. In hypertrophic cardiomyopathy, increased levels of cTnI and creatine kinase-MB (CK-MB) are associated with poorer prognoses, emphasizing their importance in predicting adverse outcomes and guiding preventive actions, such as implantable cardioverter defibrillator implantation [62]. Conversely, natriuretic peptides, including B-type natriuretic peptide (BNP) and its precursor NT-proBNP, are discharged in response to myocardial stretch and volume overload, reflecting myocardial dilation and severity of heart failure [63]. These peptides are particularly valuable in the diagnosis and treatment of heart failure as they offer additional prognostic insights beyond traditional clinical features. For example, NT-proBNP levels significantly enhance the prognostic precision of troponin models in forecasting mortality and severe complications in COVID-19 patients [58-60].

The physiological distinctions in the production, release, and breakdown of these biomarkers between males and females further complicate their clinical interpretation, highlighting the necessity for sex-specific considerations in their application [64]. Moreover, integrating these biomarkers into risk prediction models enables more accurate identification of individuals at risk of future cardiovascular events, potentially leading to earlier initiation of preventive therapies [63]. Despite their well-established roles, the specificity of troponins for MI may be diminished owing to their elevation in other myocardial conditions, emphasizing the need for further investigation to distinguish the types of troponins found in different clinical scenarios [57].

### 7.2 Emerging Biomarkers

Novel biomarkers, such as growth differentiation factor-15 (GDF-15), soluble ST2, and galectin-3, exhibit significant potential in predicting risks and diagnosing various diseases early. GDF-15, a cytokine involved in inflammation and tissue damage response, has been extensively studied and found to be elevated in numerous conditions, including non-communicable diseases, rheumatoid arthritis, juvenile dermatomyositis, and cardiovascular diseases. For example, levels of GDF-15 are notably higher in patients with rheumatoid arthritis, correlating with disease severity and cardiovascular risk, underscoring its role in inflammation and lipid metabolism [65]. Similarly, in juvenile dermatomyositis, GDF-15 levels strongly correlate with disease activity scores and functional measures, making it a valuable biomarker for evaluating disease activity and guiding treatment decisions [66]. Moreover, GDF-15 has been associated with the coronary slow flow phenomenon, where elevated levels can predict the presence and severity of the condition, highlighting its usefulness in cardiovascular diagnostics [67]. Galectin-3, an emerging biomarker, plays a critical role in inflammation, immunity, and fibrosis. It has been linked to reduced eGFR in chronic kidney disease and is implicated in renal fibrosis, making it a reliable biomarker for early detection and monitoring of kidney disease progression [68]. Galectin-3 also has potential in cardiovascular diseases as its levels can predict the severity of coronary slow flow phenomenon in conjunction with GDF-15 [65]. The use of GDF-15, soluble ST2, and galectin-3 as diagnostic tools presents a promising strategy for the early detection of diseases, categorizing risk, and enhancing patient outcomes in various medical conditions, including cardiovascular diseases.

### 7.3 Epigenetic Biomarkers

Epigenetic biomarkers have become a promising asset in the realm of cardiovascular disease (CVD) diagnosis, prognosis, and treatment, utilizing mechanisms such as DNA methylation, histone modifications, and non-coding RNAs such as microRNAs (miRNAs) and long non-coding RNAs (lncRNAs) [16,22,28,59]. Differential DNA methylation, particularly in repetitive elements and specific genomic regions, has been linked to CVD-related traits such as inflammation, dyslipidemia, hypertension, and obesity [13,17-19]. Drugs targeting DNA methyltransferases, such as hydralazine and procainamide, are being explored for their potential to hinder abnormal methylation patterns seen in CVDs [69]. Histone modifications also play a vital role in various cardiovascular conditions such as atherosclerosis, hypertension, and heart failure. Histone deacetylase inhibitors have displayed anti-proliferative and anti-inflammatory characteristics, with preclinical research affirming their cardioprotective effects [70-72]. miRNAs, which are small non-coding RNAs involved in gene regulation, have been recognized as significant contributors to the pathophysiology of several cardiovascular ailments, including myocardial infarction, coronary heart disease, and heart failure. Their presence in extracellular fluids makes them appealing circulating biomarkers with enhanced properties compared to traditional protein markers. For example, miRNAs have been suggested as diagnostic and prognostic indicators of acute coronary syndrome and other heart conditions [8,13,22].

Nonetheless, the clinical implementation of these biomarkers requires further validation through extensive multicenter studies to ensure their dependability and practicality in everyday medical practice. Progression in big data analysis and personalized epigenetic mapping is paving the way for individualized diagnosis and treatment, under the field known as pharmaco-epigenetics, which considers each patient's epigenetic foundation to forecast drug reactions and formulate personalized therapies [13, 22]. Despite the encouraging potential of epigenetic biomarkers, the transition from research to clinical use has been gradual, with ongoing trials and investigations crucial for establishing their effectiveness and safety [8,13,22]. For instance, the Bromodomain and Extra-Terminal motif inhibitor, RVX-208, demonstrated varied outcomes in trials about blood lipids, atherosclerosis, and major adverse cardiovascular events [73].

### 7.4 Metabolic Biomarkers

Metabolomic biomarkers have become an asset in the detection, prediction, and management of cardiovascular diseases (CVDs), providing thorough insight into the underlying mechanisms of the disease and assisting in tailored medical care. Essential biomarkers include natriuretic peptides (BNP/NT-proBNP) for heart failure diagnosis and prognosis evaluation and troponins for myocardial infarction diagnosis and other heart injury assessments [28,29,34,74]. Moreover, C-reactive protein is commonly used to gauge the inflammation levels linked to CVDs [75]. Lipid metabolites, such as phospholipids, sphingolipids/ceramides, glycolipids, cholesterol esters, fatty acids, and acylcarnitines, have been recognized as significant biomarkers through metabolomic profiling, with sphingolipids/ceramides displaying potential in CVD diagnosis and prognosis [76]. The amalgamation of metabolomics with genomic and proteomic information can offer a comprehensive outlook on the disease, uncovering new biochemical pathways and potential treatment targets. For example, metabolomic research has emphasized the impact of redox and nitrosative reactions on CVD progression, indicating that imbalances in these reactions can lead to disease progression. Additionally, metabolomics has played a vital role in comprehending the systemic nature of CVDs, pinpointing metabolic pathways, such as gut microbial co-metabolism, branched-chain amino acids, glycerophospholipids, cholesterol metabolism, and inflammatory processes [77]. This detailed metabolic analysis can enhance risk assessment and direct personalized therapeutic approaches, especially in complex conditions, such as coronary artery disease. Advanced technologies, such as nuclear magnetic resonance spectroscopy and liquid chromatography-mass spectrometry, have allowed the exploration of numerous metabolites, unveiling new biological pathways, and enriching our knowledge of disease development [78]. Furthermore, metabolomics has been employed to identify biomarkers linked to wholesome dietary habits that are inversely associated with CVD risk, suggesting that diet-related metabolites could function as preventive indicators [79]. Despite these encouraging prospects, challenges persist in standardizing methodologies and merging metabolomic data from various studies and platforms to ascertain clinical effectiveness. Nevertheless, continuous research and technological progress in metabolomics offers substantial potential for enhancing CVD management, ranging from early detection to individualized treatment and prevention strategies.

## 8. Challenges and Future Directions

The complexity of cardiovascular diseases (CVDs) requires unbiased approaches and incorporation of biological knowledge into computational models to improve early detection and prevention strategies. Traditional methods of detection, such as invasive angiography, have limitations that necessitate more precise and reliable solutions through machine learning and intelligent automation [42]. Progress in genomics, including whole-genome sequencing and gene-editing techniques, has created new opportunities for understanding the genetic mechanisms underlying CVDs, enabling the creation of predictive genomic models and facilitating advancements in life-course treatment and prevention. Nevertheless, the incorporation of genomics into economic health models for CVD prevention has not been fully utilized. By integrating Mendelian randomization analyses into these models, a strong economic case can be made to incorporate genomics into clinical practice, shifting our approach from reactive to preventive healthcare [80].

Machine learning models, such as neural networks, decision trees, and support vector machines, have demonstrated potential for improving the accuracy of CVD detection by analyzing diverse and complex data sources, including clinical records and omics data [38-44]. Tackling issues, such as imbalanced datasets, is crucial, as evidenced by the enhancement of the XGBoost model, which notably increased the diagnostic accuracy for heart disease [55]. Future efforts should concentrate on developing more precise and cost-efficient biomarkers for early prevention of heart disease. This entails utilizing feature selection methods, such as the relief technique, which has been proven to enhance accuracy when paired with effective classification algorithms such as the Random Forest and Extra Trees classifiers [81]. Collaborative efforts among medical professionals, data scientists, and subject matter experts are vital to guarantee the smooth integration of these innovative technologies into clinical practice, ultimately leading to a more precise, timely, and personalized diagnosis and management of CVDs [82].

## 9. Summary and Conclusion

Biomarkers play a crucial role in the early detection, diagnosis, and management of cardiovascular diseases. They provide valuable insights into the normal and abnormal conditions of the heart and vascular system, enabling timely and precise medical interventions. Commonly used biomarkers, such as high-sensitivity troponin, C-reactive protein, troponin I or troponin T, creatine kinase, B-type natriuretic peptide, and myoglobin, are essential for monitoring and treating cardiovascular diseases. Additionally, emerging biomarkers like extracellular vesicles and circular RNAs are gaining recognition for their potential in early diagnosis and treatment. Also, they play a pivotal role in understanding the pathophysiology of CVDs, risk stratification, and therapeutic decision-making. Computational biology, an interdisciplinary field, integrates various scientific disciplines to identify and validate biological markers for disease diagnosis, prognosis, and drug response prediction, thereby enhancing the diagnosis and prevention of CVDs. The integration of 'omic' data, including genomics, proteomics, and metabolomics, enables a comprehensive understanding of the molecular mechanisms underlying CVDs and facilitates the discovery of new biomarkers and treatments. Furthermore, incorporating artificial intelligence and machine learning techniques in these interdisciplinary fields is expected to enhance further our ability to analyze complex biological data and develop more accurate diagnostic and therapeutic strategies for CVDs.

## Figures and Tables

**Table 1:** Biomarkers based on different Pathophysiological processes. This table is compiled from several published reports.

Pathophysiological Process	Biomarkers
Inflammation	CRP, ST2, TNF-a, GDF-15, FAS, LP-A2, YKL-40, IL-1, Osteoprotegerin, Cytokines, Adiponectin, TREM-1, TLR-4, HMGB-1, RAGE, Danger associated molecular patterns (DAMPs), Oncostatin M, Serpins
Oxidative stress	Oxidized-LDL, Myeloperoxidase, Urinary biopyrrins, Urinary and plasma isoprostanes, urinary 8-hydroxyl-2-deoxyguanosine, Plasma malondialdehyde
Neurohormonal therapy	Norepinephrine, Renin, Angiotensin-II, Aldosterone, Arginine vasopressin, Copeptin, Endothelin −1, Urocortin, MR- proADM.
Extracellular matrix remodeling	MMP-2,3,9. TIMP1, IL-6, Collagen propeptides, N-terminal collagen type III peptide, Myostatin, Syndecan-4, Galectin-3, Oncostatin M, Serpins,
Myocyte injury	BNP, NT-proBNP, MR- proANP, sST2, GDF-15
Myocardial stretch	Troponin-T, Troponin-I, Myosin light chain kinase I, Heart-type FA binding protein, CKMB, HSP60
